# New Coarse-Grained Models to Describe the Self-Assembly of Aqueous Aerosol-OT

**DOI:** 10.1021/acs.jpcb.5c00472

**Published:** 2025-05-20

**Authors:** Alexander Moriarty, Takeshi Kobayashi, Teng Dong, Kristo Kotsi, Panagiota Angeli, Matteo Salvalaglio, Ian McRobbie, Alberto Striolo

**Affiliations:** † Department of Chemical Engineering, UCL, Gower Street, London WC1E 6BT, U.K.; ‡ Innospec Ltd., Oil Sites Road, Ellesmere Port, Cheshire, CH65 4EY, U.K.; § School of Sustainable Chemical, Biological and Materials Engineering, University of Oklahoma, Norman, Oklahoma 73019, United States

## Abstract

Aerosol-OT (AOT) is a very versatile surfactant that exhibits a plethora of self-assembly behaviors. In particular, due to its double-tail structure, it is capable of forming vesicles in water. However, the size of these structures, and the time scales over which they form, make them difficult to study using traditional all-atomistic molecular dynamics simulations. Here, three coarse-grained models are developed for AOT with different levels of detail. The models take advantage of the Martini 3 force field, which enables 2:1 mappings to be employed for the tail groups. It is shown that these models are able to reproduce the self-assembly behavior of AOT in water at three concentrations: below the critical vesicle concentration (CVC), above the CVC, and in the lamellar phase. The results also demonstrate the formation of vesicles from bicelles above the critical vesicle concentration, which is an important milestone for the continued study of vesicle behavior.

## Graphical abstract

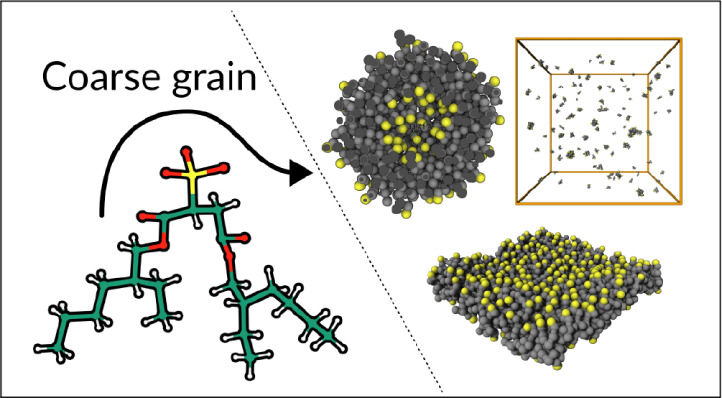

## Introduction

Aerosol-OT (sodium bis­(2-ethylhexyl) sulfosuccinate) has attracted much interest because of its ability to self-assemble into robust reverse micelles in a plethora of solvents. It has been used to study confined water behavior^1^ to template nanoparticle synthesis^2^ and to stabilize microemulsions.^3^ (We refer to Gale et al.^4^ for a review on Aerosol-OT and its applications.)

The structure of Aerosol-OT (AOT) is shown in Figure 1. It has been suggested that the “cone-like” molecular structure of AOT is responsible for its phase behavior, but Nave et al.^3^ argue that this is a myth; AOT simply has a convenient phase boundary around room temperature, and other sulfosuccinates are just as good at stabilizing microemulsions, but at different temperatures or using different oils.^3,5–7^

**Figure 1:**
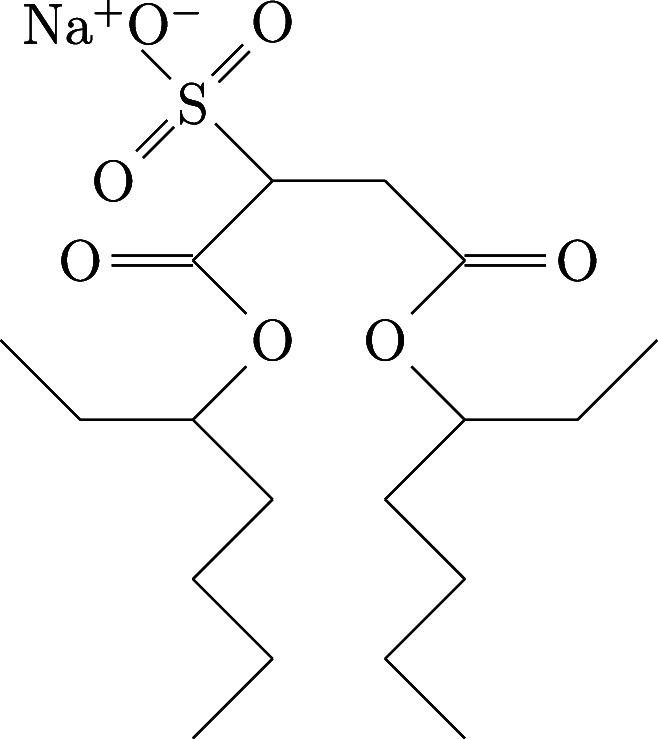
Skeletal structure of Aerosol-OT.

One thing that each of the sulfosuccinate surfactants have in common is their doubled tail groups. According to the packing parameter theory of Israelachvili,^8^ such surfactants lend themselves to forming vesicles. AOT is no exception; indeed, it has a characteristic critical vesicle concentration (CVC) of 7.5 mM in water, above which its micelles transition into vesicles.^9^

Vesicles have been studied extensively in a biological context, as they play an essential function in several physiological processes^10^ and they show promise in enhancing drug delivery systems.^11^ More recently, another important application of vesicles has been discovered: the control of wetting and bouncing behavior of droplets. Cryo-TEM images of aqueous systems containing AOT above and below the CVC demonstrate that vesicular AOT facilitates a wetting transition on hydrophobic leaf surfaces, thereby reducing droplet bounce.^12^

To elucidate the mechanisms responsible for these observations, and clarify whether vesicles are indeed essential for these types of phenomena, it is desirable to couple experiments such as those in the literature with molecular simulations. In fact, because of its wide range of applications, AOT has attracted the interest of many computational studies, which have been, for the most part, conducted at the atomistic level.^13–20^

Vesicles are relatively large self-assembled structures, whose size is highly variable. For example, Fan et al.^9^ reported an average radius of AOT vesicles in water at 10 mM of approximately 150 nm, as obtained using dynamic light scattering (DLS) and transmission electron microscopy (TEM) measurements, while Velázquez et al.^21^ reported a radius of 40 nm under the same conditions using cryo-TEM. Irrespective of the exact radius, the size range observed experimentally imposes the use of large simulation boxes and a very large number of self-assembling structures, should one desire to observe the formation of vesicles during the course of unbiased simulations. Because of the related computational constraints, coarse-grained (CG) models are required, rather than atomistic ones.

Coarse-grained models allow one to reduce the number of particles in a molecular dynamics simulation by treating groups of atoms as a single unit (a *bead*). The bead represents the collective motion of the group of atoms, as well as the net interaction with other beads. The scaling of the number of particles with the simulation dimensions is significantly reduced by coarse-graining, allowing larger systems to be studied. Furthermore, because the degrees of freedom are reduced, CG simulations can study longer time scales compared to atomistic ones within the same computational time. However, care must be taken to ensure the models are accurate.

Here, we develop a CG model for AOT and show that it is capable of reproducing experimental self-assembly behavior. While the level of detail achieved with our method is less than atomistic models,^17–20^ the coarse-grained model presented here is capable of reproducing several experimental observables such as bilayer thickness and the transition of micelles to vesicles as the surfactant concentration increases.

## Method

To enhance the atomistic models available in the literature, our goal is to develop a realistic coarse-grained model for AOT. For this purpose, we used the recently released Martini 3 force field to establish bead interaction parameters, as it boasts more accurate predictions of molecular packing than its predecessor,^22^ as well as the option to include varying ratios of atom-to-bead mapping, which enables chemically similar molecules to be distinguished when a 4-to-1 mapping would be imprecise.^23^ Furthermore, the Martini 3 force field provides more detailed models of ions through the use of beads of different sizes.

Others have attempted to derive coarse-grained models for AOT surfactants. For example, Negro et al.^24^ developed one such model using Martini 2.^25^ However, the authors admit that the model is “somewhat generic” because the two tail group beads could represent any alkyl chain between 6 to 10 carbons long. Because the tail groups have a pronounced effect on behavior^3,5^ it is desirable to develop a model that is coarse grained enough to provide economy of computational power, but detailed enough to allow us to study the effect of variations of the molecular architecture on its properties.

In our approach, CGBuilder^26^ was used to map atoms to coarse-grained beads. Three mappings were generated using different scales of mapping for the carbon tails: Coarsest uses exclusively 4:1 mappings (R beads), Finest uses 2:1 mappings (T beads) and Mixed uses a combination. The resulting mappings are shown in Figure 2. Bead types were selected based on the suggestions by Souza et al.^23^

**Figure 2:**
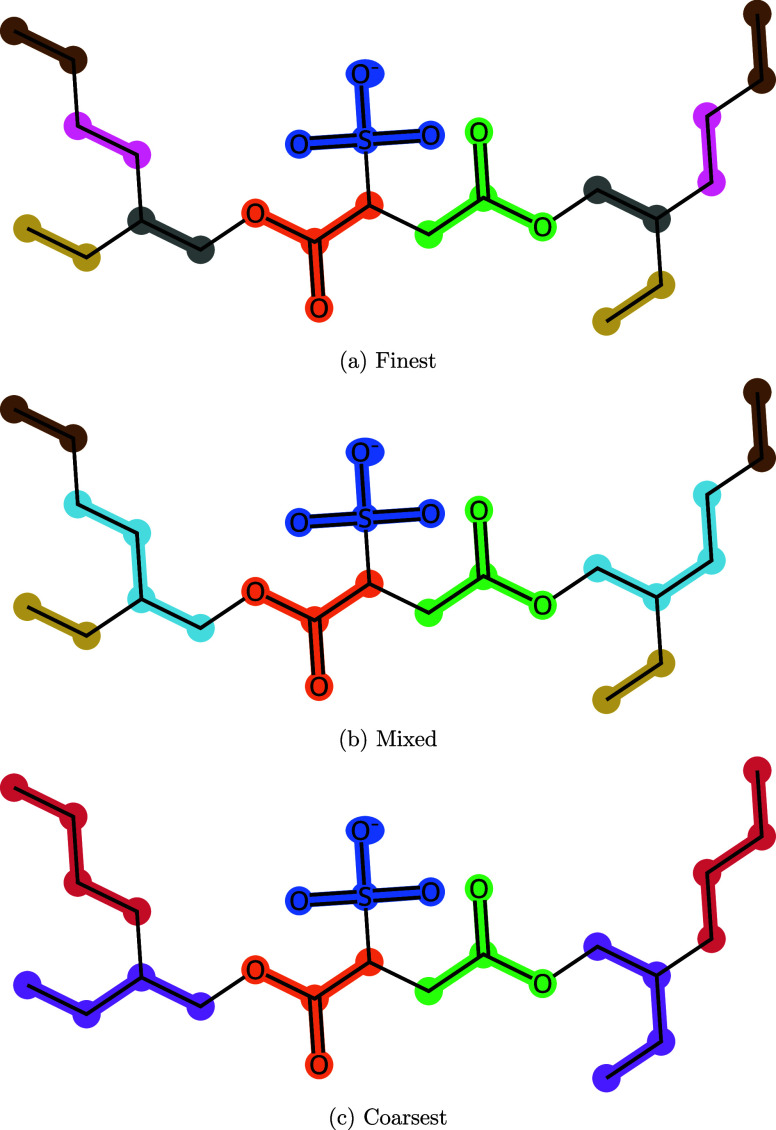
Coarse-grained mappings of AOT at different resolutions: (a) Finest, (b) Mixed and (c) Coarsest. Sets of highlighted atoms and bonds are mapped to a single bead. Symmetrically equivalent beads are highlighted in the same colors; for a given mapping, bonds between pairs of beads of the same colors have the same parameters. For example, the bonded parameters between the red and purple tail groups of the Coarsest model are identical.

Simulations of a single AOT molecule in water were performed for each of the three mapping schemes. The distribution of bond lengths and angles in the resulting trajectories were compared to a 50 ns all-atomistic (AA) simulation of the same system using the force field developed by Abel et al.^20^ The AA trajectory was mapped to an effective coarse-grained (CG) trajectory by determining the center-of-geometry (mean coordinates) of the constituent atoms for each bead.

The bonded parameters must be fit to the bond length and angle distributions from the AA trajectory. This optimization was achieved using SwarmCG.^27^ SwarmCG implements swarm optimization coupled with Boltzmann inversion to fit the bonded parameters to the AA target distribution. Boltzmann inversion has been successfully employed in parametrizing CG models, such as in the case of nanoparticles^28^ and swarm optimization acts to efficiently fine-tune the parameters. Because of the symmetry of the AOT molecule, symmetrically equivalent bonds in the tails were assigned the same parameters and optimized jointly (see Figure 2).

In the Martini family of force fields, it is standard to exclude nonbonded interactions between directly bonded particles.^29^ However, in AOT both carboxylate beads are close to the sulfonate group, but only one of them is bonded to it. The nonbonded, intramolecular interaction between these beads caused simulation instability and led to a poor match in the bond angle distribution between the three groups. The nonbonded, intramolecular interactions between the sulfonate and both carboxylate beads were therefore excluded. The resulting bond angle and bond length distributions are shown in the Supporting Information. The resulting topology files, in GROMACS ITP format,^30^ are available in the Supporting Information.

### Validation

Because we are ultimately interested in studying vesicles, which are self-assembled structures, it is pertinent to validate the models based on their self-assembly behavior. To that end, three sets of simulations were performed to study the properties of different types of structures: bilayer, micelles, and vesicles.

#### Dilute Isotropic Phase

To study the properties of self-assembled micelles and vesicles, systems of 1 and 0.27 wt % AOT in water were generated for each CG model. These systems were created by randomly dispersing AOT molecules in a cubic box of water. The number of molecules in each system and the initial box dimensions are given in Table 1.

**Table 1:** Number of AOT Molecules and Water Beads for the Coarse-Grained AOT Simulations^a^

Model	Concentration (wt % AOT)	Number of AOT	Number of Water Beads	Initial Box Size (nm)
Finest	0.27	200	452043	38
	1.0	200	128903	25.2
	18.0	300	8453	—
Mixed	0.27	200	453128	38
	1.0	180	111937	24
	18.0	340	9667	—
Coarsest	0.27	200	453348	38
	1.0	180	112134	24
	18.0	350	9965	—

a The dimensions of the bilayer systems were semi-isotropic and are reported in the Bilayers section.

After setting up the initial configuration, the following procedure was used for the coarse-grained simulations:

1. An energy minimization step using steepest descent integration, for 100000 steps or until the maximum force in the system was no greater than 100 kJ mol^–1^ nm ^–1^.
2. For the 0.27 wt % systems only, an NVT simulation was conducted to increase the temperature from 0 K to 298 K across 5 ns (Δ*t* = 10 fs).
3. An NPT equilibration for 10 fs (Δ*t* = 10 fs) using a stochastic cell rescaling barostat^31^ (τ = 4 ps).
4. Finally, a production run using an NPT ensemble (Δ*t* = 20 fs) using a Parrinello–Rahman barostat^32^ (τ = 12 ps). The 1 wt % systems were run for 2.5 μs, while the 0.27 wt % systems were run for 23 μs.

For each of steps 2 to 4, a V-rescale thermostat^33^ (τ = 1 ps) was used to control the temperature. For the 0.27 wt % systems, the solvent has many more degrees of freedom than the solute and therefore it is more sensitive to stochastic velocity rescaling. This makes these systems susceptible to the “hot-solvent, cold-solute” effect when coupling the entire system using one thermostat, whereby the average temperature of the solute is much lower than that of the solvent.^34–36^ To overcome this artifact, it was necessary to thermostat the AOT and the solvent (water and Na^+^ ions) separately for the 0.27 wt % systems.

In order to characterize the aggregates formed during the simulations, a clustering algorithm was applied to the final production run. If the distance between two surfactant tail group beads was smaller than a threshold value, *r*_cut_, the parent molecules were considered to be part of the same aggregate. This threshold value was set to $\frac{5}{4}\sigma_{\mathrm{LJ}}$ , where σ_LJ_ is the distance at which the Lennard-Jones potential between the smallest tail group beads is zero (*r*_cut_ = 5.875 Å for the Coarsest model and *r*_cut_ = 4.250 Å for the Mixed and Finest models). This choice of threshold respects the difference in the equilibrium distance between the tail groups of the different models, and was empirically observed to yield reasonable results.

Several properties of the aggregates were calculated. The radius of gyration, *R*_g_, was determined using

1 $$R_{g}=\sqrt{\frac{\overset{N}{\underset{i=1}{\sum }}m_{i}{\mathrm{r⃗}}_{i}^{2}}{\overset{N}{\underset{i=1}{\sum }}m_{i}}}$$

where *N* is the number of surfactants in the aggregate, *r⃗*_*i*_ is the displacement of the *i*th surfactant from the center of mass of the aggregate and *m_i_* is the mass of the *i*th surfactant.

The Willard–Chandler surface^37^ of each aggregate was calculated using the Pytim^38^ and PyVista^39^ Python packages. The kernel density estimate of each aggregate’s surfactants’ beads was computed using a Gaussian kernel with a bandwidth of 4.0 Å. This can be thought of as a smoothing parameter. If it is too high, the geometry of the aggregate’s surface may be lost. If it is too low, voids may appear in the surface, corresponding to regions of lower density within the aggregate.

The code was amended to accelerate the computation of the density estimate using JAX.^40^ Rather than using the minimum image convention, a supercell of 3 × 3 × 3 was created and the kernel was evaluated using all positions within this supercell. This allowed the use of JAX’s GPU acceleration when calculating the density estimate, as it does not support periodic boundary conditions and instead requires the input coordinates to exist in linear space.

The density estimate was then evaluated on a grid with spacing 2.0 Å. The Willard–Chandler isosurface was then generated using a density cutoff of $\frac{\rho_{\max}}{3}$ , where ρ_max_ was the maximum computed density for the aggregate. The enclosed volume of the surface is negligibly larger (approximately 5% on average) using the JAX-accelerated method, but the computation time is improved significantly. The choice of cutoff and bandwidth were found empirically to yield surfaces that were very close to the locations of the headgroups, while avoiding artifacts (like gaps in the surface) that could be caused by fluctuations of the density within the core of the micelles. The volume and surface area of each Willard–Chandler surface were calculated to determine the volume and surface area per surfactant of each aggregate.

Following the procedure of Gale et al.,^4^ the coordinate-pair eccentricities (CPEs) of the aggregates were also determined. An ellipsoid has three principal axes, *a* ≥ *b* ≥ *c*. The moments of inertia of the ellipsoid are related to the principal axes by

2 $$A=\frac{M}{5}\left(b^{2}+c^{2}\right)$$

3 $$B=\frac{M}{5}\left(a^{2}+c^{2}\right)$$

4 $$C=\frac{M}{5}\left(a^{2}+b^{2}\right)$$

The principal axes can therefore be solved from the moments of inertia algebraically.

In order to characterize this shape without losing information, two eccentricity values can be calculated:

5 $$e_{ab}=\sqrt{1-\frac{b^{2}}{a^{2}}}$$

6 $$e_{ac}=\sqrt{1-\frac{c^{2}}{a^{2}}}$$

Depending on the lengths of the three principal axes, and therefore the eccentricity values, four types of ellipsoids can be characterized:

Spherical ellipsoids have *a* ≈ *b* ≈ *c* and *e*_*ab*_ ≈ *e*_*ac*_ ≈ 0.

Prolate ellipsoids have *a* ≫ *b* ≈ *c* and *e*_*ab*_ ≈ *e*_*ac*_ ≫ 0.

Oblate ellipsoids have *a* ≈ *b* ≫ *c* and *e*_*ab*_ ≪ *e*_*ac*_ ≫ 0.

Triaxial ellipsoids have *a* *b* ≫ *c* and *e*_*ab*_ < *e*_ac_ ≫ 0.

These different types of ellipsoids can be distinguished graphically by plotting the CPE values; see Figure 3 for a schematic example.

**Figure 3:**
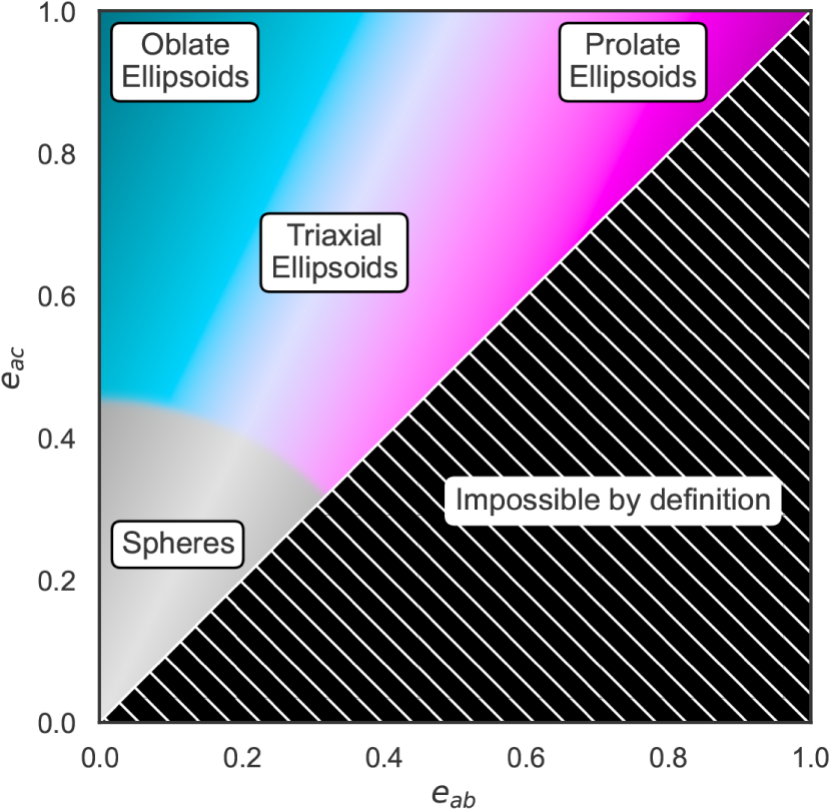
Illustration of the coordinate-pair eccentricity (CPE) values for different types of ellipsoids. Adapted from Gale et al.^4^ Copyright 2022 American Chemical Society.

Each of these properties was calculated in 2 ns intervals across the simulation trajectory for aggregates with aggregation number ≥5. The simulations were considered equilibrated once these values had reached a steady state. As discussed in the Dilute isotropic phasein Results section, for some systems it was not possible to achieve a steady state despite sampling across several microseconds.

In order to assess the convergence of the simulations, pyMSER^41^ was used to analyze the timeseries of the mean system properties, averaged across simultaneously coexisting aggregates. That is, the properties were calculated for each aggregate at each time step, then reduced to one mean system property per time step, for each of the following: volume per surfactant in aggregate, surface area per surfactant in aggregate, radius of gyration, and aggregation number. The evolution of these mean properties was compared across simulation time. pyMSER implements an algorithm to determine the optimal truncation point, *k*, for timeseries data in order to minimize the marginal standard error (MSE) of the remaining data. The MSE is defined as

7 $$\mathrm{MSE}\left(k\right)=\frac{1}{{\left(n-k\right)}^{2}}\overset{n-1}{\underset{i=k}{\sum }}{\left(Y_{i}-{\mathrm{Y̅}}_{n,k}\right)}^{2}$$

where *n* is the total number of timesteps, *Y*_*i*_ is the value of the property at time step *i*, and Y̅ _*n*,*k*_ is the mean of the property from time step *k* to *n*. Intuitively, the prefactor (*n* – *k*)^−2^ biases the metric toward maximizing the remaining sample size, therefore improving confidence, while the sum term penalizes the deviation of the remaining data from the mean. The data from *k* to *n* is then used to calculate the equilibrium mean and standard deviation.

In addition, pyMSER employs the augmented Dickey–Fuller test to verify that the remaining data is stationary.^42^ Briefly, this method tests the null hypothesis that a unit root is present in the data, which would indicate that the data is nonstationary. The test statistic is compared to a critical value, which is dependent on the number of observations and the significance level. If the test statistic is less than the critical value, the null hypothesis is rejected and the data is considered stationary. For each of the dilute isotropic phase systems, convergence was tested by confirming that the equilibrium data for the aforementioned properties was stationary with at least 99% confidence.

We note that the geometrical properties measured here do not necessarily reflect internal molecular rearrangements within the aggregates, which have been shown to be significant particularly in the case of multicomponent systems with large amphiphiles, such as those found in biological systems.^43–47^ However, there is insufficient experimental data regarding the internal conformation of AOT aggregates to validate the behavior of the models in this respect. Furthermore, because AOT is a comparatively small surfactant, and the tail groups are chemically homogeneous, it is expected that the conformational space within an aggregate is limited.

#### Bilayers

The properties of spontaneously assembled bilayers were studied to compare to experimental data. A system of 18 wt % AOT in water was created for each CG model, which is well above the lamellar phase transition of aqueous AOT at 298 K.^48^

Because the mapping ratio is different for each model, so too is their molecular volume. During the solvation step, each bead was given the same van der Waals radius, so that the coarser models occupied a smaller volume and water was packed more densely around them in the initial configuration. This difference in density was corrected during the pressure coupling stage.

The number of molecules in the starting configurations are given in Table 1. It is important to note that the mass of the beads in the Martini parametrization is not necessarily equal to the total mass of the atoms that they are supposed to represent; the mass depends only on the size of the bead, i.e., how many non-hydrogen atoms it represents, regardless of their atomic masses.^23^ The concentrations reported here were determined by converting the mole fractions using the *real* molar mass of AOT (*M*_r_ = 444.6).

To simplify the analysis, the system was extended in the *z*-axis, so that it was more likely the bilayer would form in the *xy*-plane. Specifically, a cubic box (10 nm sides) of randomly dispersed AOT was solvated in a rectangular box of solvent (10 nm × 10 nm × 15 nm).

The simulation procedure was as follows:

1. An energy minimization stage, for 100000 steps or until the maximum force in the system was no greater than 100 kJ mol ^–1^ nm^–1^.
2. An NPT ensemble, using isotropic pressure coupling, for 50 ns (Δ*t* = 20 fs). This was in order to allow a bilayer to form in the *xy*-plane. A stochastic cell rescaling barostat (τ = 4 ps, κ = 3 × 10^–4^ bar^–1^)^31^ was used to couple the pressure.
3. A further 150 ns (Δ*t* = 20 fs) NPT simulation using a semi-isotropic stochastic cell rescaling barostat (τ = 4 ps, κ_*xy*_ = κ_*z*_ = 3 × 10^–4^ bar^–1^).

A stochastic velocity rescaling thermostat was used to control the temperature^33^ (τ = 1 ps, *T* = 298 K). We found that using only one thermostat was sufficient to control the temperature. This is likely because the concentration of surfactants is relatively large and therefore the difference in the number of degrees of freedom between the solute and solvent is less significant, so that the temperature of the solvent and solute remain the same.

During the semi-isotropic pressure coupling stage, the *xy*-area of the simulation box changes in dimension in order to minimize the in-plane tension of the bilayer. After this area had converged, two properties were measured: bilayer thickness and area-per-headgroup.

The Willard–Chandler surface of the bilayer was computed using the method described in the Dilute Isotropic Phase section. In this case there are two layers: one upper and one lower. The marching cubes algorithm used to identify the Willard–Chandler surface does not automatically partition the vertices into these layers.^49^ For these calculations, the Pyvista software library^39^ was used to identify which vertices belong in which layer based on their connectivity. This yields two surfaces (one for each layer).

The area per headgroup was calculated by dividing the combined area of the two surfaces by the number of surfactants, while the thickness was determined by calculating the nearest-neighbor distance between the two surfaces at each vertex.

## Results and Discussion

### Dilute Isotropic Phase

Figure 4 shows the normalized aggregation numbers for each dilute isotropic system as a function of simulation time. Each of the 1 wt % systems forms a monolithic aggregate after approximately 1 μs, while the 0.27 wt % systems exhibit a much more gradual growth and present a distribution of aggregation numbers at equilibrium.

**Figure 4:**
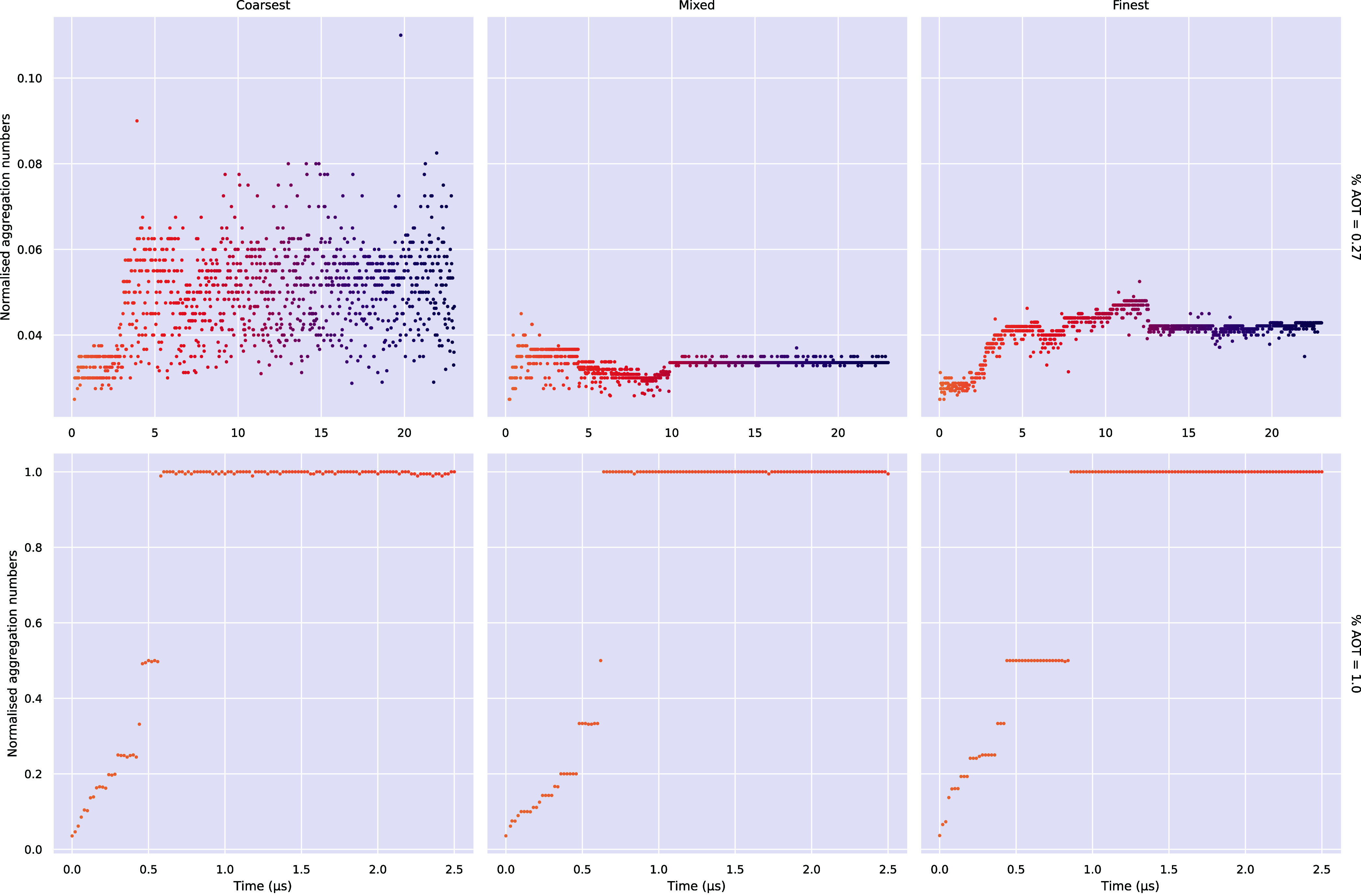
Normalized aggregation numbers (fraction of total number of surfactants in a cluster) for each aggregate (aggregation number ≥5) in the dilute isotropic systems. Only aggregates with an aggregation number of at least 5 are shown.

It is important to note that the types of aggregates obtained differ depending on the surfactant concentration. For each model, the 1 wt % systems form a *vesicle* shortly after the surfactants have assembled into a monolithic aggregate, while the 0.27 wt % systems form micelles; see Figure 5 for a snapshot of each of the Mixed model systems in their final configurations.

**Figure 5:**
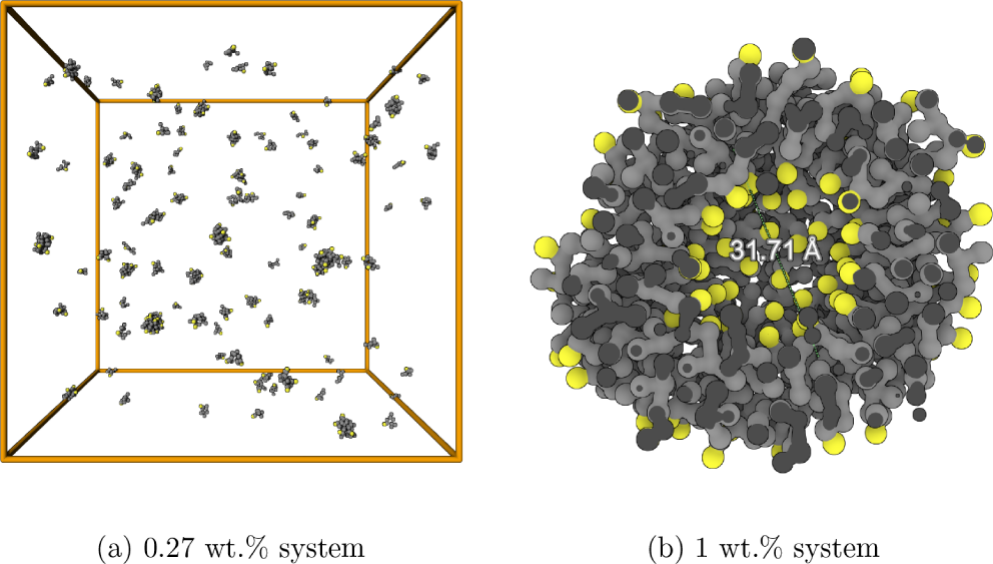
Final configurations of the Mixed dilute isotropic systems at (a) 0.27 wt % and (b) 1 wt % AOT. Yellow beads represent surfactant headgroups and gray beads represent tail groups. The box in 5­(a) is isotropic, of side ≈ 37 nm.

Several studies report that the critical vesicle concentration (CVC) of AOT occurs at 7.5 mM, which is approximately 0.33 wt %.^9,21,50^ By definition, above this concentration the surfactants spontaneously self-assemble into vesicles. Our results are consistent with these data; we observe micelles below the CVC and vesicles above it.

For completeness, it should be noted that some studies have used small-angle neutron scattering (SANS) to measure micelle properties at concentrations well above the CVC.^51–53^ Furthermore, prior all-atomistic molecular dynamics simulations found not vesicles, but micelles at 1 wt % AOT in water.^19^ It is possible that micelles coexist alongside vesicles at these concentrations, though the results of Velázquez et al.^21^ suggest that the population of micelles must be very small.

We also note that, as discussed in the Introduction, the dynamics of coarse-grained simulations are generally much faster than for all-atomistic simulations. For our Coarsest 1 wt % system, vesicle formation took approximately 600 ns. A typical scaling factor to convert the effective time from a coarse-grained to all-atomistic system is around 4,^25^ which implies that the transition may take more than 2 μs to occur when all-atomistic simulations are used to simulate the self-assembly process. This is longer than the runtime used by Bhat et al.,^19^ which may explain why they did not observe vesicle formation despite having considered concentrations above the CVC.

Figure 6 shows the evolution of the properties of the aggregates in the 1 wt % systems. Visual analysis of these results suggests that, for our models, there is an increase in the time taken to form a vesicle as the degree of coarse-graining decreases. This is explained by the fact that the potential energy surface becomes rougher as the degrees of freedom increase. Therefore, the dynamics of the Finest model are much slower than those of the Coarsest model.

**Figure 6:**
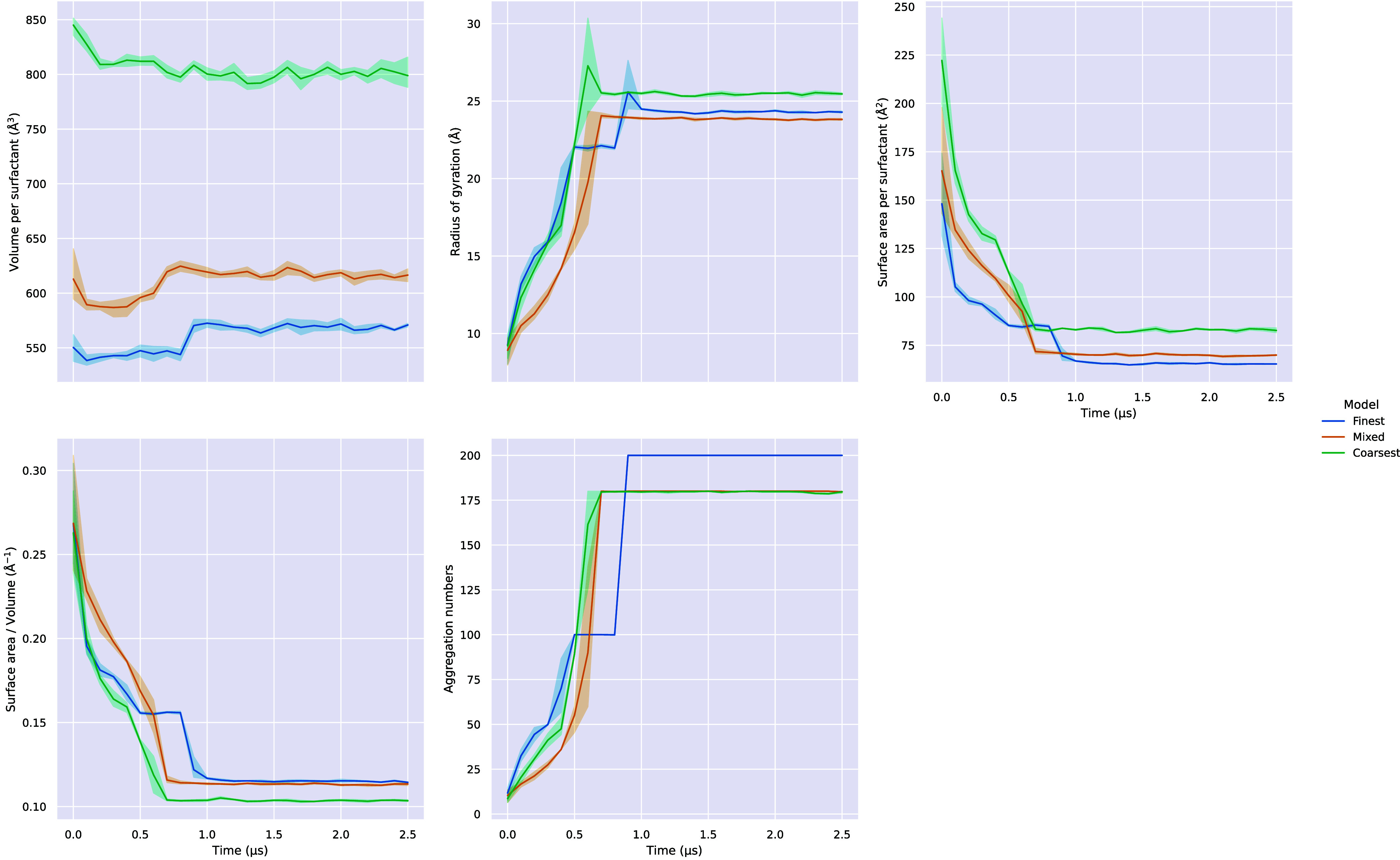
Mean surface area to volume ratio, surface area per surfactant, volume per surfactant, and aggregation number of aggregates (aggregation number ≥5) in 100 ns bins for each of the 1 wt % systems. Shaded area represents the 95% confidence interval calculated using bootstrapping.

The size and shape of the vesicles is very consistent between our models. The main difference is that there is a monotonic increase in the surface area and volume per surfactant as the degree of coarse-graining increases. This implies that the more coarsely grained models are unable to pack as densely, which we attribute to the larger average van der Waals radius of the beads. This is consistent with the results obtained for the bilayer simulations, as discussed later.

We note that the size of the vesicles is likely constrained by the box size and the number of surfactants in the simulation. In order to establish the equilibrium size of a vesicle, larger simulations must be performed. This will be the subject of a future paper. Some researchers find that the average hydrodynamic radius of AOT 10 mM vesicles is approximately 40 nm 70 nm,^21^ while others find it may be as large as 150 nm.^9^ Considering that vesicles are likely dynamic systems not necessarily at equilibrium, this difference in reported size of vesicles is not surprising. Nevertheless, it should be pointed out that these values exceed the size of the simulation boxes that can be considered using our current computational resources.

Notably, there is a brief jump in aggregate volume in Figure 6 once the surfactants have formed a monolithic aggregate. This jump corresponds to a transitional period, before the aggregate becomes a vesicle. Analysis of the simulation snapshots at this point reveals the presence of disk-like structures, which are referred to as “bicelles” in the literature.^54^ They are essentially like small bilayers, in that they are planar, but they do not percolate across the periodic boundaries of the simulation box.

During this transition, the bicelle begins to curve into a concave shape, until finally the edges meet to form a vesicle. This process is well described in the literature and has been observed experimentally for other surfactants^55–58^ but to our knowledge it is the first time this process has been demonstrated for simulations of AOT. This suggests that our CG models, coupled with the long simulation times implemented in this work, are appropriate for identifying key steps in the self-assembly of AOT. Snapshots from this transition are shown for the Mixed system in Figure 7 and a video showing the full transition is available in the Supporting Information.

**Figure 7:**
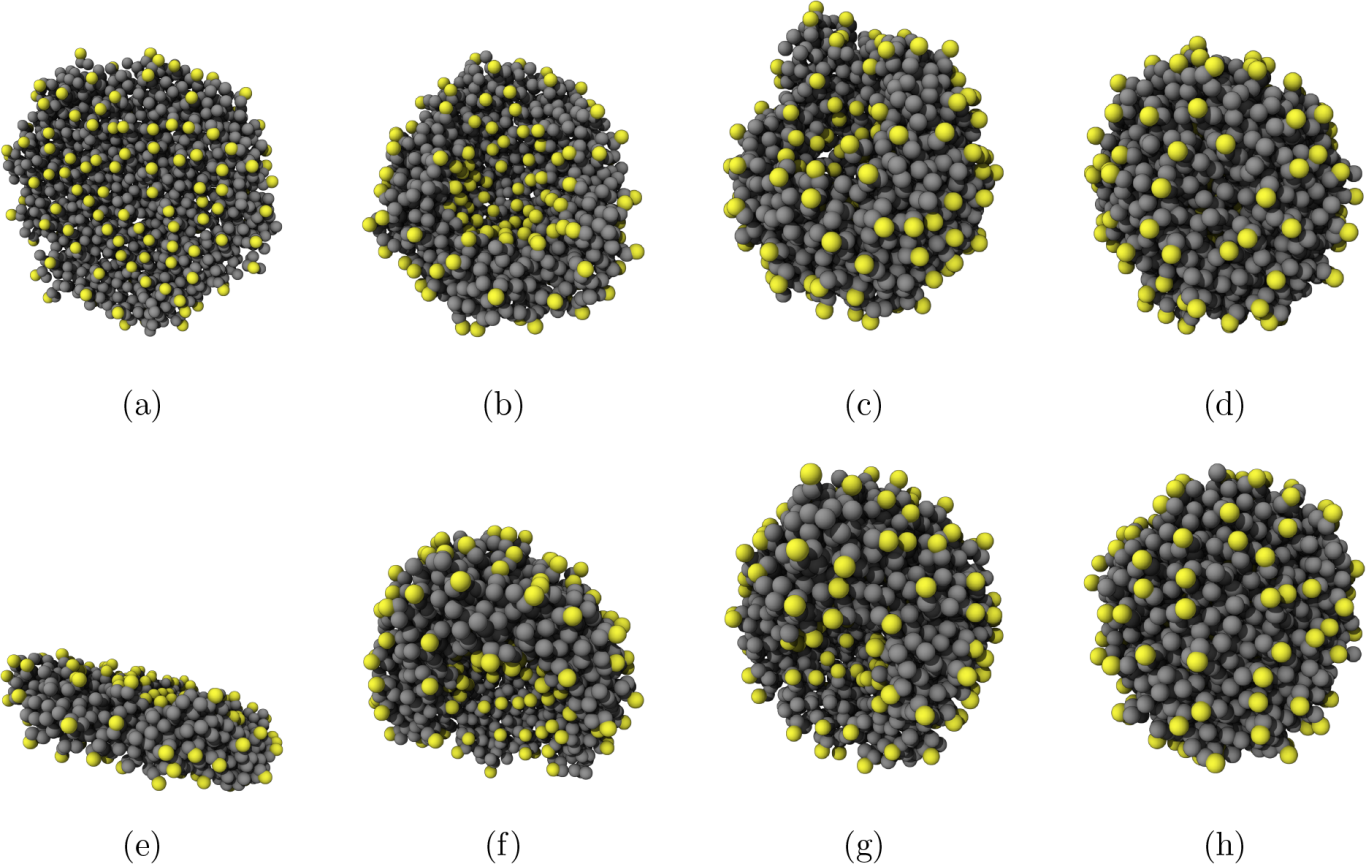
Stages of bicelle-to-vesicle transition for the Mixed model. The initial bicelle (a/e) begins to curve in on itself (b,c/f,g), eventually closing to become a vesicle (d/h). The top row shows the side view and the bottom row shows the top view at the same time step.

Figure 8 shows the evolution of aggregate properties for the 0.27 wt % systems. These are much larger than the 1 wt % systems, and the growth process observed in our simulations is not monotonic; surfactants will often leave the aggregates. This highlights their metastable nature. Because of these competing processes, it takes far longer to reach equilibrium. The stochastic nature of the growth process suggests that it may be necessary to repeat the simulations several times in order to more reliably characterize the micelles. Furthermore, despite the fact that these simulations are quite large, they are still subject to finite size effects. Fajalia et al.^51^ report that the mean intermicellar distance for this concentration of AOT in water is 223.2 Å, which is much larger than the maximum possible distance in our simulation boxes.

**Figure 8:**
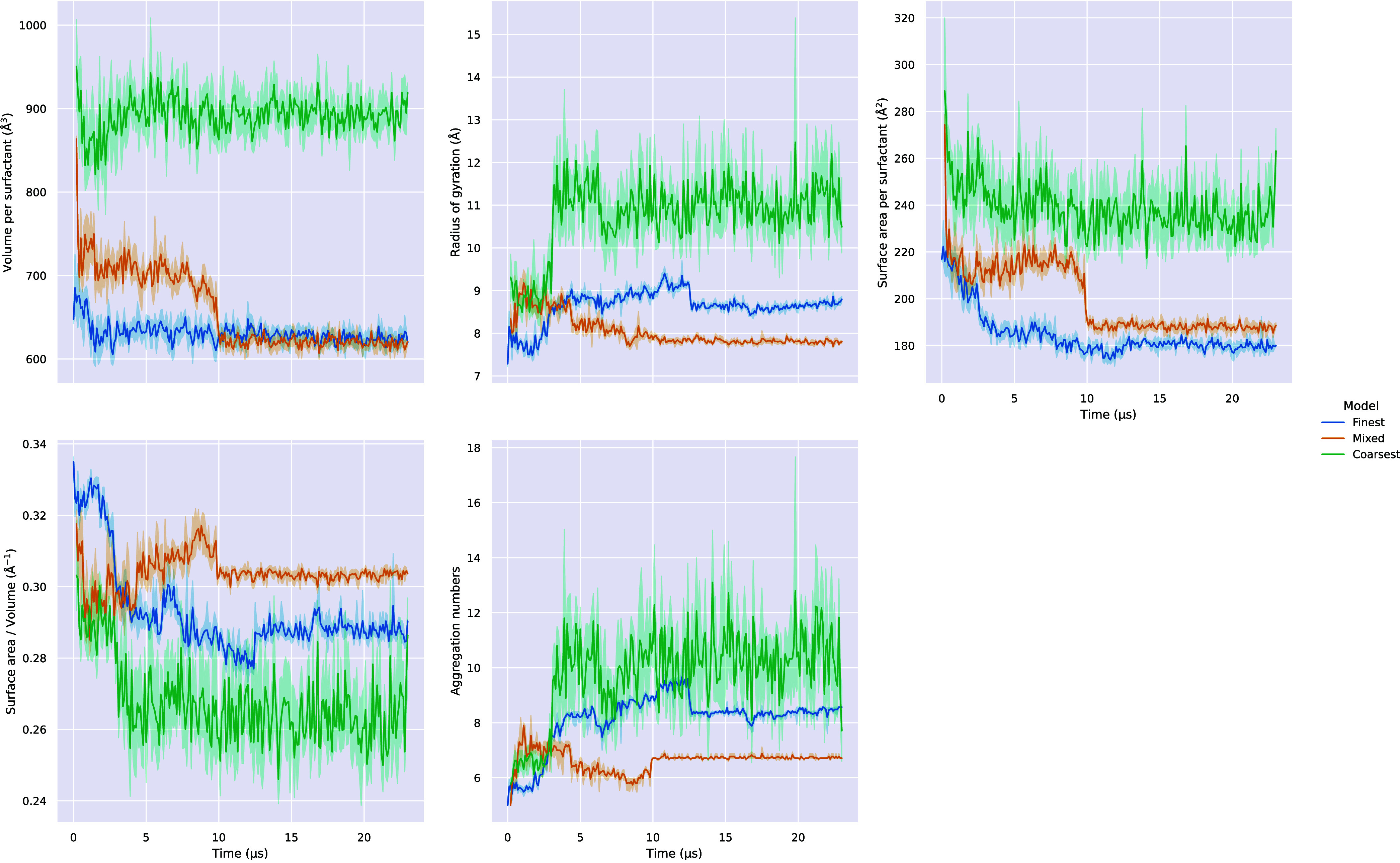
Mean surface area to volume ratio, surface area per surfactant, volume per surfactant, and aggregation number of aggregates (aggregation number ≥5) in 100 ns bins for each of the 0.27 wt % systems. Shaded area represents the 95% confidence interval calculated using bootstrapping.

At 0.27 wt %, although the Mixed and Finest systems’ properties appear to reach a plateau, with only one or two coexisting micelles, the Coarsest system exhibits a much greater degree of polydispersity and the properties fluctuate a lot more, even once converged. This is likely due to the faster dynamics that are characteristic of the Coarsest model, which facilitate surfactant exchange across shorter time scales. The large variance in aggregate properties across the equilibrium regime is also explained by the metastable nature of the aggregates. In any case, the simulation results show that, qualitatively, the three models yield very consistent results.

The resulting time-averaged mean property values and their standard deviation are reported in Table 2. The experimental value for the radius of gyration, *R*_g_, was calculated using the experimentally determined ratio of the major to minor semiaxes, ε = 1.22:^51^

8 $$R_{g}=\frac{c}{\sqrt{5}}\sqrt{1+2\varepsilon^{2}}$$

**Table 2:** Time-Averaged Mean Properties of the Systems (Averaged across Aggregates with Aggregation Number ≥5) in the 0.27 Wt % Simulations Compared to Experimental Values^a^

Model	Final Convergence Time (μs)	Mean Surface Area to Volume Ratio (Å^–1^)	Mean Surface Area per Surfactant (Å^2^)
Finest	12.6	0.288 ± 0.004	180 ± 4
Mixed	10.3	0.303 ± 0.002	188 ± 3
Coarsest	3.08	0.265 ± 0.015	238 ± 16
Experiment^51^	–	0.15	194.3

Model	Mean Aggregation Number	Mean Radius of Gyration (Å)
Finest	8.4 ± 0.1	8.6 ± 0.1
Mixed	6.7 ± 0.1	7.8 ± 0.1
Coarsest	10.2 ± 0.2	11.0 ± 0.1
Experiment^51^	22.8	11

a Convergence times were evaluated using pyMSER^41^ by first taking the mean of each property across the aggregates at each timestep, then analyzing the timeseries of these values. The convergence time for each property was different; the final convergence time is the maximum of these values. The uncertainty reported here is the standard deviation of the mean (averaged across simultaneous aggregates) property measured across the converged region. The experimental radius of gyration was calculated using eq 8.

For consistency, eq 8 employs the same assumptions made by Fajalia et al:^51^ that the micelles are oblate ellipsoids (*a* ≈ *b* ≫ *c*), with a semiaxis *c* = 12.57 Å, corresponding to the length of a fully stretched alkyl tail. Fajalia et al.^51^ used these assumptions to infer the volume of their micelles. Our results suggest that these assumptions may not be accurate in all conditions, as our values for CPE indicate that the micelles are predominantly prolate or triaxial ellipsoids. Care must be taken, therefore, when comparing the computational values of *R*_g_ and the surface area to volume ratio to the experimental ones. For completeness, we note that in both of these cases, the Coarsest model is in closer agreement with the experimental values. This correlates with the fact that the Coarsest model exhibited the largest average aggregation number.

The surface area per surfactant for the Mixed and Finest models seems to be in very good agreement with the experimental value of 194.3 Å^–2^. We consider the CG models derived here reasonable approximations of reality, although one should be careful in extrapolating results obtained for the size of AOT micelles.

Finally, Figure 9 shows the coordinate-pair eccentricities (CPEs) of every aggregate across the trajectories of the simulations. The vesicles, which correspond to a normalized aggregation number of 1, are all mostly spherical. Conversely, micelles are almost always prolate ellipsoids. Generally, larger micelles have a greater *e*_*ac*_ than smaller micelles. The transitional bicelles can also be identified in the CPE plots for the 1 wt % systems: they are the points with a normalized aggregation number of 1 that exhibit a very high *e*_*ac*_ value and an intermediate *e*_*ab*_ value, indicating that they are triaxial ellipsoids.

**Figure 9:**
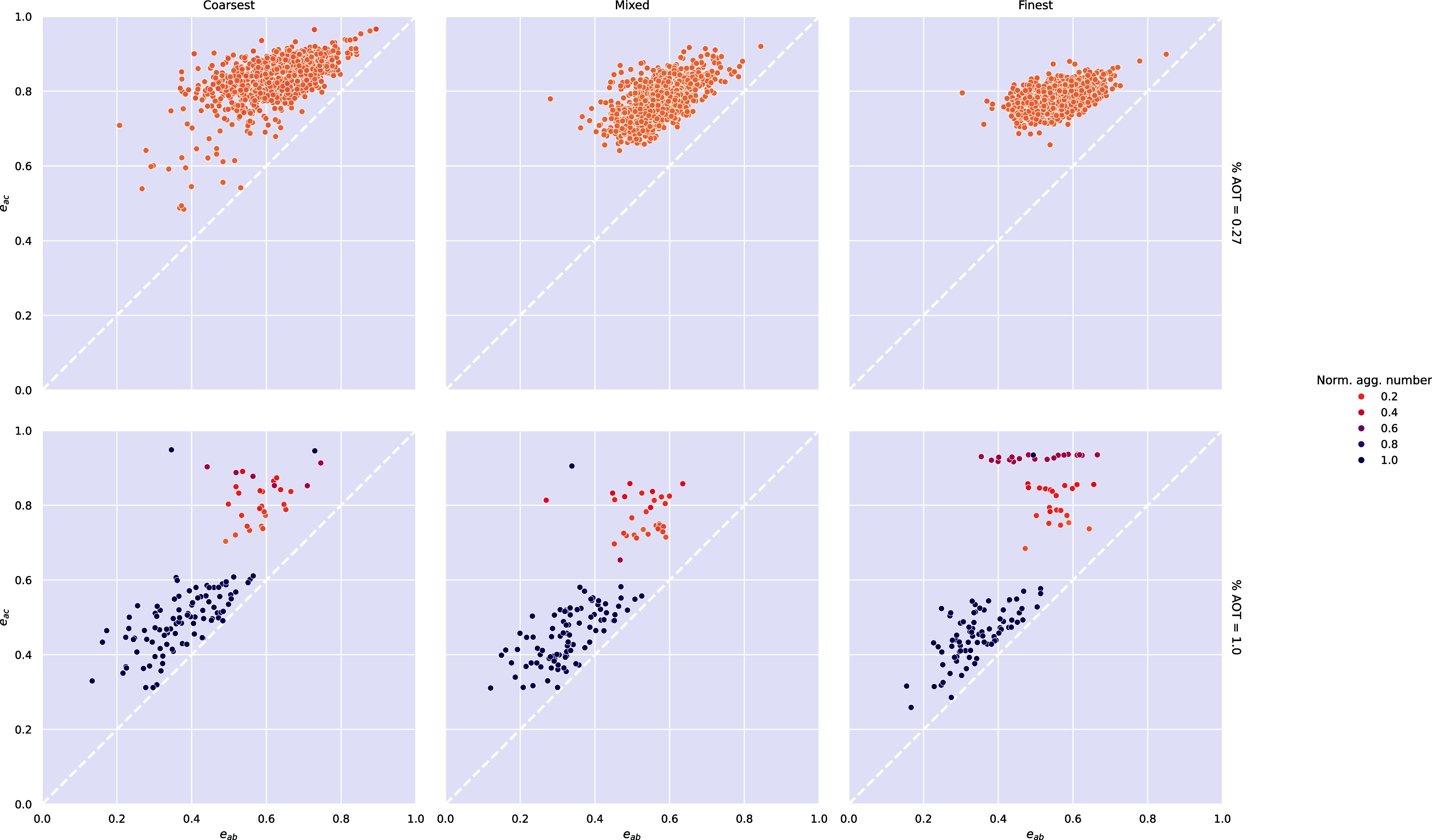
Coordinate-pair eccentricities of every aggregate (aggregation number ≥5) across the entire trajectory for each of the coarse-grained simulations. See also Figure 3 for a schematic interpretation of this mapping.

### Bilayer Properties

To assess the convergence of the bilayer simulations, the *xy*-area of the simulation box was monitored. After 30 ns, all of the systems’ areas had converged. Notably, there was a monotonic increase in the equilibrium area and the time taken to reach it as the degree of coarse-graining increased. This is due to the fact that the coarser systems were initialized with more AOT molecules. The timeseries is shown in the Supporting Information.

The distributions of the bilayer thickness measurements and the area per headgroup were calculated for time *t* > 30 ns for each system. The results are shown in Figure 10 and the mean values are reported in Table 3. The thicknesses obtained from the simulations using the Finest and Mixed models are quite similar; both are ∼5% thinner than the experimental value. The Coarsest model bilayer, however, is over 10% thicker on average than the experimental data.

**Figure 10:**
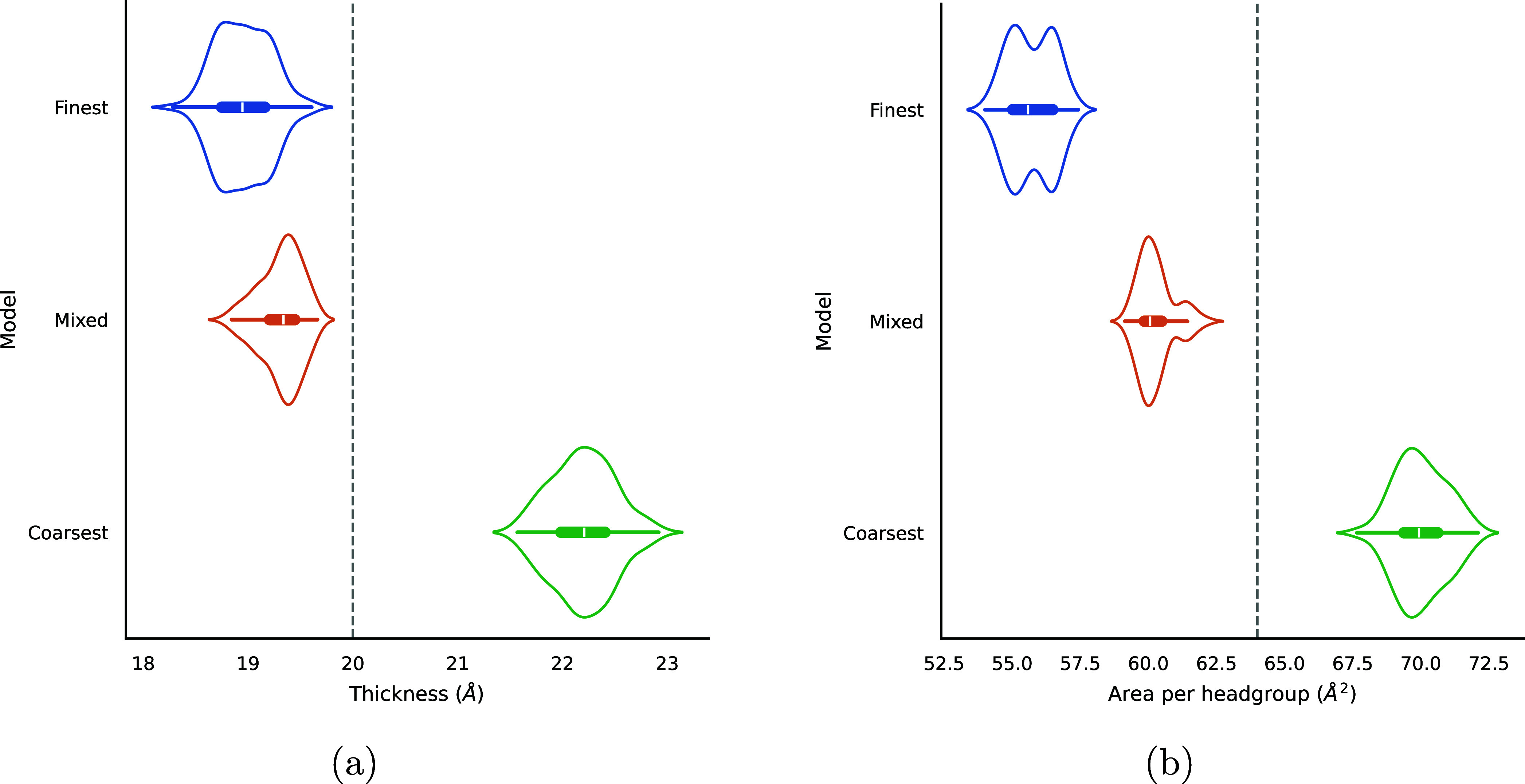
Combined kernel density estimate and box-and-whisker plots showing the distributions of (a) thickness and (b) area per headgroup of the AOT bilayer for each CG model. The distributions are aggregated across *t* > 30 ns. Dashed lines indicate experimental values.^59^ Experimental values were derived by Fontell^59^ using X-ray diffraction.

**Table 3:** Mean Bilayer Thickness and Area Per Headgroup for Each CG Model

Model	Thickness (Å)	Area per headgroup (Å^2^)
Finest	18.96	55.71
Mixed	19.32	60.25
Coarsest	22.20	70.00
Experiment^59^	20.0	64.0

There is a more significant difference in the area per headgroup. In this case, the Mixed and Coarsest models are both within ∼10% of the experimental value. The Finest model’s mean area per headgroup is ∼13% lower than the experimental value. The area per headgroup exhibits a monotonic increase with respect to the degree of coarse-graining. This mirrors the trend observed for the dilute isotropic systems, whereby the more coarsely grained models are unable to pack as densely.

Based on these results, the Mixed model seems to be the most consistent with the experimental data, though all of the models derived here are reasonably accurate. This is in spite of the fact that the Finest model is “closer” to the atomistic model in terms of the number of degrees of freedom. This may be due to the way that Martini was parametrized: the finer degrees of mapping are only recommended when necessary, at the end of chains or in the case of branched chains, where a 4-to-1 mapping represents too large of a group.^23^

## Conclusions

In this work, three coarse-grained models for AOT were developed with different degrees of mapping from the atomistic scale: Finest, Mixed and Coarsest. The models were validated by simulating systems of different concentrations; two systems (0.27 and 1.0 wt %) below the solubility limit of AOT in water, which represent the dilute isotropic phase, and one (18 wt %) which represents an anisotropic phase, in order to study the properties of self-assembled bilayers.

It was found that all of the models were able to broadly reproduce the expected self-assembly behavior of AOT in water, forming micelles at 0.27 wt % (below the critical vesicle concentration) and vesicles at 1.0 wt % (above the CVC). The 0.27 wt % system was characterized by very slow dynamics, with the Finest model requiring up to 13 μs to converge. The systems at higher concentrations converged faster. Of note, the simulations at 1.0 wt % showed, for the first time, in the case of AOT, the transition from the large, disc-like micelles known as “bicelles” to vesicles. The vesicles were found to be spherical, while the micelles were prolate ellipsoidal.

There are two principal differences in the behavior of the models. First, the more coarsely grained models are unable to pack as densely, which is reflected in the surface area per surfactant and the volume of the vesicles, as well as the area-per-headgroup of the bilayers. Second, the dynamics of the models are faster for the more coarsely grained systems, as evidenced by the rate of micelle growth.

The Mixed model appears to be the most reliable choice based on the agreement between our simulations and experimental bilayer properties. This model promises a new way to interrogate the behavior of AOT vesicles on the molecular scale and, simultaneously, to study their effect on macroscopic behavior like droplet bouncing and spreading. This will be the subject of future work.

## Supplementary Material





## Data Availability

Scripts to reproduce the simulations and analyses performed here can be found on GitHub at https://github.com/a-ws-m/aot-cg-simulations and https://github.com/a-ws-m/aot-analysis.

## Notes

The topology and structure files (in GROMACS ITP/GRO format) for the CG models; animations of the vesicle formation processes for each of the CG models (ZIP)
Snapshots of the final configurations of each of the dilute isotropic and bilayer systems, and the Willard–Chandler surface of the bilayers; *xy*-area timeseries for the bilayer simulations; bond and angle distributions for a single molecule of AOT in water compared between the all-atomistic and coarse-grained models (PDF)

## References

[1] Levinger N. E. (2002). Water in
Confinement. Science.

[2] Spirin M. G., Brichkin S. B., Razumov V. F. (2005). Synthesis
and Stabilization of Gold
Nanoparticles in Reverse Micelles of Aerosol OT and Triton X-100. Colloid J..

[3] Nave S., Eastoe J., Heenan R. K., Steytler D., Grillo I. (2000). What Is So
Special about Aerosol-OT? 2. Microemulsion Systems. Langmuir.

[4] Gale C. D., Derakhshani-Molayousefi M., Levinger N. E. (2022). How to Characterize
Amorphous Shapes: The Tale of a Reverse Micelle. J. Phys. Chem. B.

[5] Nave S., Eastoe J., Penfold J. (2000). What Is So Special
about Aerosol-OT?
1. Aqueous Systems. Langmuir.

[6] Nave S., Eastoe J., Heenan R. K., Steytler D., Grillo I. (2002). What Is So
Special about Aerosol-OT? Part III. Glutaconate versus Sulfosuccinate
Headgroups and Oil-Water Interfacial Tensions. Langmuir.

[7] Nave S., Paul A., Eastoe J., Pitt A. R., Heenan R. K. (2005). What Is
So Special about Aerosol-OT? Part IV. Phenyl-Tipped Surfactants. Langmuir.

[8] Israelachvili, J. N. Intermolecular and Surface Forces; Academic press, 2011.

[9] Fan Y., Li Y., Yuan G., Wang Y., Wang J., Han C. C., Yan H., Li Z., Thomas R. K. (2005). Comparative Studies on the Micellization
of Sodium Bis­(4-Phenylbutyl) Sulfosuccinate and Sodium Bis­(2-Ethylhexyl)
Sulfosuccinate and Their Interaction with Hydrophobically Modified
Poly­(Acrylamide). Langmuir.

[10] György B., Szabó T. G., Pásztói M., Pál Z., Misják P., Aradi B., László V., Pállinger É., Pap E., Kittel Á. (2011). Membrane Vesicles, Current State-of-the-Art: Emerging
Role of Extracellular Vesicles. Cell. Mol. Life
Sci..

[11] Vader P., Mol E. A., Pasterkamp G., Schiffelers R. M. (2016). Extracellular
Vesicles for Drug Delivery. Adv. Drug Delivery
Rev..

[12] Song M., Ju J., Luo S., Han Y., Dong Z., Wang Y., Gu Z., Zhang L., Hao R., Jiang L. (2017). Controlling Liquid
Splash on Superhydrophobic Surfaces by a Vesicle Surfactant. Sci. Adv..

[13] Poghosyan A. H., Adamyan M. P., Shahinyan A. A., Koetz J. (2019). AOT Bilayer Adsorption
on Gold Surfaces: A Molecular Dynamics Study. J. Phys. Chem. B.

[14] Poghosyan A. H., Adamyan M. P., Shahinyan A. A. (2019). A Rippled
Defective Phase of AOT
Lamella: A Molecular Dynamics Study. Colloids
Surf., A.

[15] Aminian A., ZareNezhad B. (2019). Oil-Detachment
from the Calcium Carbonate Surfaces
via the Actions of Surfactant, Nanoparticle and Low Salinity Brine:
An Insight from Molecular Dynamic Simulation. Chem. Eng. Sci..

[16] Urano R., Pantelopulos G. A., Straub J. E. (2019). Aerosol-OT Surfactant
Forms Stable
Reverse Micelles in Apolar Solvent in the Absence of Water. J. Phys. Chem. B.

[17] Honegger P., Steinhauser O. (2019). Towards Capturing Cellular Complexity:
Combining Encapsulation
and Macromolecular Crowding in a Reverse Micelle. Phys. Chem. Chem. Phys..

[18] Schmollngruber M., Braun D., Oser D., Steinhauser O. (2016). Dielectric
Depolarisation and Concerted Collective Dynamics in AOT Reverse Micelles
with and without Ubiquitin. Phys. Chem. Chem.
Phys..

[19] Bhat A., Harris M. T., Jaeger V. W. (2021). Structural
Insights into Self-Assembled
Aerosol-OT Aggregates in Aqueous Media Using Atomistic Molecular Dynamics. J. Phys. Chem. B.

[20] Abel S., Sterpone F., Bandyopadhyay S., Marchi M. (2004). Molecular Modeling
and Simulations of AOT-Water Reverse Micelles in Isooctane: Structural
and Dynamic Properties. J. Phys. Chem. B.

[21] Velázquez M. M., Valero M., Ortega F., Rodríguez González J. B. (2007). Structure
and Size of Spontaneously Formed Aggregates in Aerosol OT/PEG Mixtures:
Effects of Polymer Size and Composition. J.
Colloid Interface Sci..

[22] Marrink S. J., Risselada H. J., Yefimov S., Tieleman D. P., de Vries A. H. (2007). The MARTINI
Force Field: Coarse Grained Model for Biomolecular Simulations. J. Phys. Chem. B.

[23] Souza P. C. T., Alessandri R., Barnoud J., Thallmair S., Faustino I., Grünewald F., Patmanidis I., Abdizadeh H., Bruininks B. M. H., Wassenaar T. A. (2021). Martini 3: A General Purpose Force Field for Coarse-Grained Molecular
Dynamics. Nat. Methods.

[24] Negro E., Latsuzbaia R., de Vries A. H., Koper G. J. M. (2014). Experimental
and Molecular Dynamics Characterization of Dense Microemulsion Systems:
Morphology, Conductivity and SAXS. Soft Matter.

[25] Marrink S. J., de Vries A. H., Mark A. E. (2004). Coarse
Grained Model for Semiquantitative
Lipid Simulations. J. Phys. Chem. B.

[26] Barnoud, J. Jbarnoud/Cgbuilder. Cgbuilder, 2024.

[27] Empereur-Mot C., Pesce L., Doni G., Bochicchio D., Capelli R., Perego C., Pavan G. M. (2020). Swarm-CG: Automatic
Parametrization of Bonded Terms in MARTINI-Based Coarse-Grained Models
of Simple to Complex Molecules via Fuzzy Self-Tuning Particle Swarm
Optimization. ACS Omega.

[28] Chan E. R., Striolo A., McCabe C., Cummings P. T., Glotzer S. C. (2007). Coarse-Grained
Force Field for Simulating Polymer-Tethered Silsesquioxane Self-Assembly
in Solution. J. Chem. Phys..

[29] Alessandri, R. ; Thallmair, S. ; Herrero, C. G. ; Mera-Adasme, R. ; Marrink, S. J. ; Souza, P. C. T. A Practical Guide to Recent Advances in Multiscale Modeling and Simulation of Biomolecules; Wang, Y. ; Zhou, R. AIP Publishing LLC, 2023; pp. 1–34.

[30] Abraham M. J., Murtola T., Schulz R., Páll S., Smith J. C., Hess B., Lindahl E. (2015). GROMACS: High Performance
Molecular Simulations through Multi-Level Parallelism from Laptops
to Supercomputers. SoftwareX.

[31] Bernetti M., Bussi G. (2020). Pressure Control Using
Stochastic Cell Rescaling. J. Chem. Phys..

[32] Parrinello M., Rahman A. (1981). Polymorphic Transitions in Single Crystals: A New Molecular
Dynamics Method. J. Appl. Phys..

[33] Bussi G., Donadio D., Parrinello M. (2007). Canonical
Sampling through Velocity
Rescaling. J. Chem. Phys..

[34] Mor A., Ziv G., Levy Y. (2008). Simulations
of Proteins with Inhomogeneous Degrees
of Freedom: The Effect of Thermostats. J. Comput.
Chem..

[35] Lingenheil M., Denschlag R., Reichold R., Tavan P. (2008). The “Hot-Solvent/Cold-Solute
Problem Revisited. J. Chem. Theory Comput..

[36] Cheng A., Merz K. M. (1996). Application of the
Nosé-Hoover Chain Algorithm
to the Study of Protein Dynamics. J. Phys. Chem..

[37] Willard A. P., Chandler D. (2010). Instantaneous Liquid
Interfaces. J. Phys. Chem. B.

[38] Sega M., Hantal G., Fábián B., Jedlovszky P. (2018). Pytim: A Python
Package for the Interfacial Analysis of Molecular Simulations. J. Comput. Chem..

[39] Sullivan C. B., Kaszynski A. A. (2019). PyVist: 3D Plotting and Mesh Analysis through a Streamlined
Interface for the Visualization Toolkit (VTK). J. Open Source Softw..

[40] Jax-Ml/Jax. jax -ml, Jax, 2024.

[41] Oliveira F. L., Luan B., Esteves P. M., Steiner M., Neumann
Barros Ferreira R. (2024). pyMSER-An Open-Source Library for Automatic Equilibration
Detection in Molecular Simulations. J. Chem.
Theory Comput..

[42] Greene, W. H. Econometric Analysis; Pearson Education India, 2003.

[43] Ziolek R. M., Smith P., Pink D. L., Dreiss C. A., Lorenz C. D. (2021). Unsupervised
Learning Unravels the Structure of Four-Arm and Linear Block Copolymer
Micelles. Macromolecules.

[44] Cardellini A., Crippa M., Lionello C., Afrose S. P., Das D., Pavan G. M. (2023). Unsupervised Data-Driven
Reconstruction of Molecular
Motifs in Simple to Complex Dynamic Micelles. J. Phys. Chem. B.

[45] Gardin A., Perego C., Doni G., Pavan G. M. (2022). Classifying
Soft
Self-Assembled Materials via Unsupervised Machine Learning of Defects. Commun. Chem..

[46] Cho Y., Christoff-Tempesta T., Kaser S. J., Ortony J. H. (2021). Dynamics in Supramolecular
Nanomaterials. Soft Matter.

[47] Iyer J., Mendenhall J. D., Blankschtein D. (2013). Computer Simulation–Molecular-Thermodynamic
Framework to Predict the Micellization Behavior of Mixtures of Surfactants:
Application to Binary Surfactant Mixtures. J.
Phys. Chem. B.

[48] Rogers J., Winsor P. A. (1967). Optically Positive, Isotropic and Negative Lamellar
Liquid Crystalline Solutions. Nature.

[49] van
der Walt S., Schönberger J.
L., Nunez-Iglesias J., Boulogne F., Warner J. D., Yager N., Gouillart E., Yu T. (2014). Scikit-Image: Image Processing in Python. PeerJ.

[50] Briz J. I., Velázquez M. M. (2002). Effect
of Water-Soluble Polymers on the Morphology
of Aerosol OT Vesicles. J. Colloid Interface
Sci..

[51] Fajalia A. I., Antoniou E., Alexandridis P., Tsianou M. (2015). Self-Assembly of Sodium
Bis­(2-Ethylhexyl) Sulfosuccinate in Aqueous Solutions: Modulation
of Micelle Structure and Interactions by Cyclodextrins Investigated
by Small-Angle Neutron Scattering. J. Mol. Liq..

[52] Sheu E. Y., Chen S. H., Huang J. S. (1987). Structure
and Growth of Bis­(2-Ethylhexyl)
Sulfosuccinate Micelles in Aqueous Solutions. J. Phys. Chem..

[53] Sheu E. Y., Chen S. H. (1988). Thermodynamic Analysis of Polydispersity in Ionic Micellar
Systems and Its Effect on Small-Angle Neutron Scattering Data Treatment. J. Phys. Chem..

[54] Sanders C. R., Prosser R. S. (1998). Bicelles: A Model Membrane System for All Seasons?. Structure.

[55] Sut T. N., Yoon B. K., Park S., Jackman J. A., Cho N.-J. (2020). Versatile
Formation of Supported Lipid Bilayers from Bicellar Mixtures of Phospholipids
and Capric Acid. Sci. Rep..

[56] Yue B., Huang C.-Y., Nieh M.-P., Glinka C. J., Katsaras J. (2005). Highly Stable
Phospholipid Unilamellar Vesicles from Spontaneous Vesiculation: A
DLS and SANS Study. J. Phys. Chem. B.

[57] Leng J., Egelhaaf S. U., Cates M. E. (2002). Kinetic
Pathway of Spontaneous Vesicle
Formation. Europhys. Lett..

[58] Leng J., Egelhaaf S. U., Cates M. E. (2003). Kinetics of the
Micelle-to-Vesicle
Transition: Aqueous Lecithin-Bile Salt Mixtures. Biophys. J..

[59] Fontell K. (1973). The Structure
of the Lamellar Liquid Crystalline Phase in Aerosol OT—Water
System. J. Colloid Interface Sci..

