# Supplementary material for: New Coarse-Grained Models to Describe the Self-Assembly of Aqueous Aerosol-OT
Source: J Phys Chem B. 2025 May 20;129(21):5299–310. doi: 10.1021/acs.jpcb.5c00472 (PMC12128036; doi:10.1021/acs.jpcb.5c00472)
Supplement: Supplementary file 1 [file jp5c00472_si_001.zip › si/si.pdf]

Supplementary Information to *New Coarse-Grained Models to Describe the Self-Assembly of Aqueous Aerosol-OT*

January 21, 2025

S1 Bond and angle distributions

Figures S1, S2 and S3 show the distributions of bond lengths and angles within a single molecule of AOT in water across 10 ns using different coarse-grained models. For comparison, the same distributions are shown for a 40 ns simulation using an all-atomistic model [1]. These graphs were generated by SwarmCG [2].

The bond and angle group indexes correspond to those in the topology files (in GROMACS ITP format): at the end of each bond and angle definition, there is a comment (after the `;`) with the index of the group to which the bond or angle belongs. Where two bonds or angles are defined with the same group index, they are considered to be equivalent by symmetry. These groups have the same parameters and the distribution shown in the graph represents the combined observations of every bond/angle in the group. Note that the bond and angle groups are separate: bond group 1 does not necessarily correspond to angle group 1.

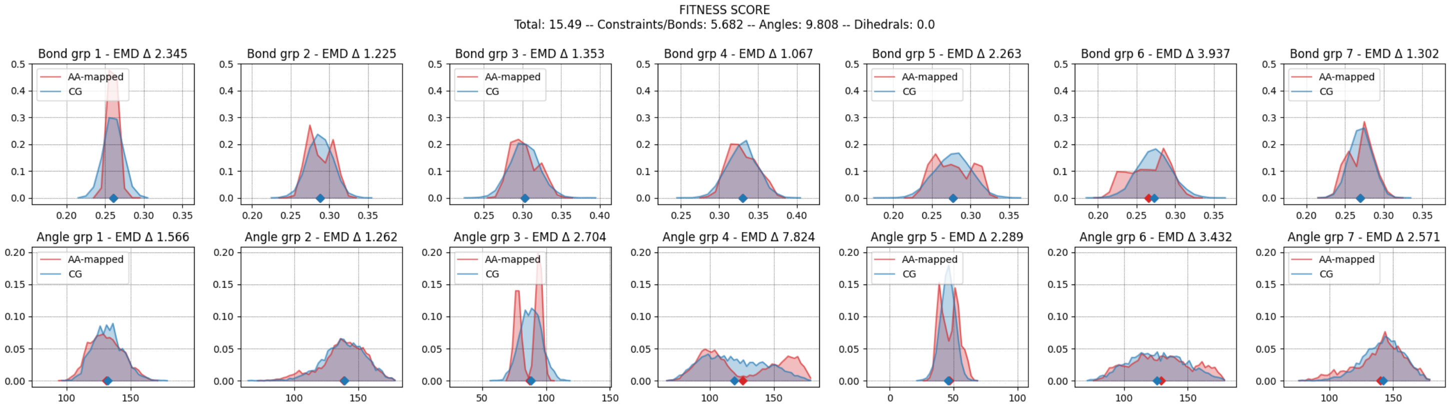

Figure S1: Bond length and angle distributions for the Finest model compared to the same distributions for the all-atomistic model.

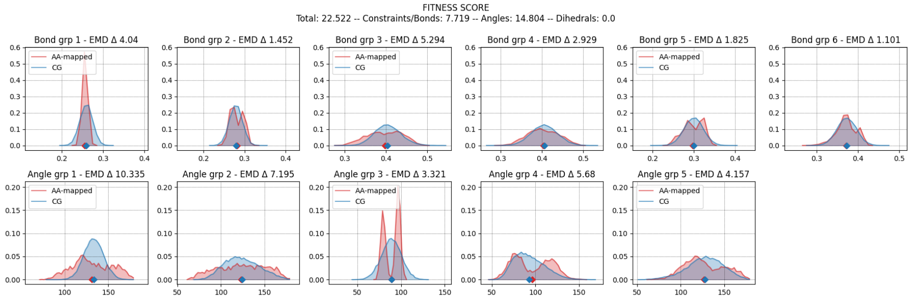

Figure S2: Bond length and angle distributions for the Mixed model compared to the same distributions for the all-atomistic model.

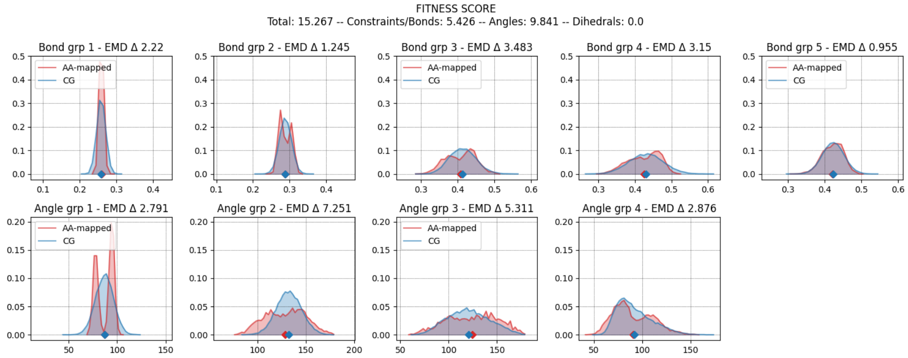

Figure S3: Bond length and angle distributions for the Coarsest model compared to the same distributions for the all-atomistic model.

S2 Dilute isotropic configurations

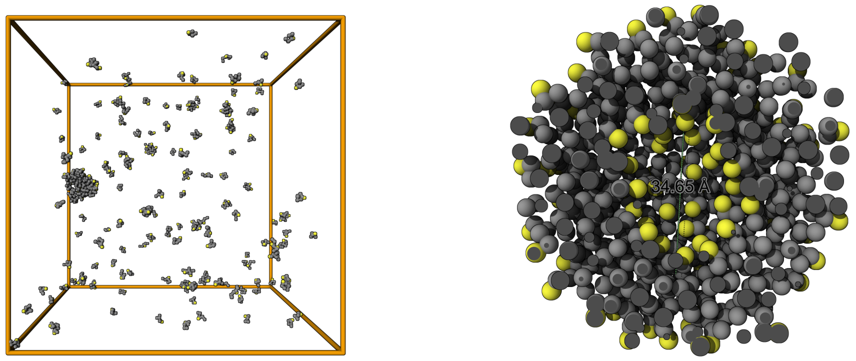

(a) 0.27 wt.% system

(b) 1 wt.% system

Figure S4: Final configurations of the Coarsest dilute isotropic systems. Yellow beads represent surfactant headgroups and grey beads represent tail groups. The box in S4a is isotropic, of side ≈ 37 nm.

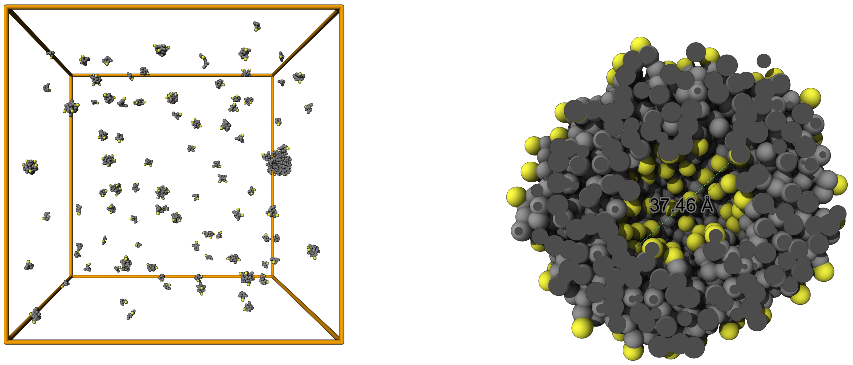

(a) 0.27 wt.% system

(b) 1 wt.% system

Figure S5: Final configurations of the Finest dilute isotropic systems. Yellow beads represent surfactant headgroups and grey beads represent tail groups. The box in S5a is isotropic, of side ≈ 37 nm.

S3 Final bilayer configurations

Figure S6 shows the final configurations of the Mixed and Finest bilayer simulations, alongside the Willard-Chandler surface, computed using the Method described in the main paper.

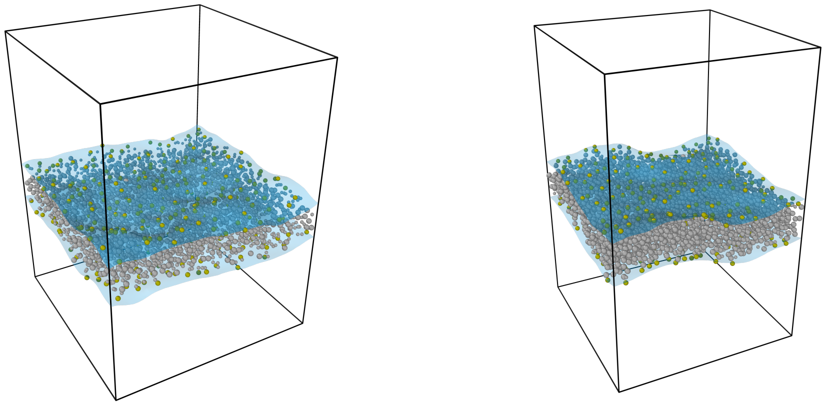

(a) Mixed model

(b) Finest model

Figure S6: Final configurations of the Mixed and Finest bilayer simulations. Yellow beads represent surfactant headgroups and grey beads represent tail groups. The computed Willard-Chandler surface is highlighted in blue.

References

[1] Stéphane Abel et al. "Molecular Modeling and Simulations of AOT-Water Reverse Micelles in Isooctane: Structural and Dynamic Properties". In: *The Journal of Physical Chemistry B* 108.50 (Dec. 2004), pp. 19458–19466. ISSN: 1520-6106. DOI: 10.1021/jp047138e. (Visited on 06/08/2023).

[2] Charly Empereur-Mot et al. "Swarm-CG: Automatic Parametrization of Bonded Terms in MARTINI-Based Coarse-Grained Models of Simple to Complex Molecules via Fuzzy Self-Tuning Particle Swarm Optimization". In: *ACS Omega* 5.50 (Dec. 2020), pp. 32823–32843. DOI: 10.1021/acsomega.0c05469. (Visited on 05/03/2024).
