# Supplementary material for: New Coarse-Grained Models to Describe the Self-Assembly of Aqueous Aerosol-OT
Source: J Phys Chem B. 2025 May 20;129(21):5299–310. doi: 10.1021/acs.jpcb.5c00472 (PMC12128036; doi:10.1021/acs.jpcb.5c00472)
Supplement: Supplementary file 2 [file jp5c00472_si_002.pdf]

New Coarse-Grained Models to Describe the Self-Assembly of Aqueous Aerosol-OT

Supporting Information

Alexander Moriarty[1], Takeshi Kobayashi[1], Teng Dong[1], Kristo Kotsi[1], Panagiota Angeli[1], Matteo Salvalaglio[1], Ian McRobbie[2], and Alberto Striolo[3]

[1]Department of Chemical Engineering, UCL, Gower Street, London WC1E 6BT, UK

[2]Innospec Ltd., Oil Sites Road, Ellesmere Port, Cheshire, CH65 4EY, UK

[3]School of Sustainable Chemical, Biological and Materials Engineering, University of Oklahoma, Norman, OK 73019, USA

S1 Bond and angle distributions

Figures S1, S2 and S3 show the distributions of bond lengths and angles within a single molecule of AOT in water across 10 ns using different coarse-grained models. For comparison, the same distributions are shown for a 40 ns simulation using an all-atomistic model [1]. These graphs were generated by SwarmCG [2].

The bond and angle group indexes correspond to those in the topology files (in GROMACS ITP format): at the end of each bond and angle definition, there is a comment (after the ;) with the index of the group to which the bond or angle belongs. Where two bonds or angles are defined with the same group index, they are considered to be equivalent by symmetry. These groups have the same parameters and the distribution shown in the graph represents the combined observations of every bond/angle in the group. Note that the bond and angle groups are separate: bond group 1 does not necessarily correspond to angle group 1.

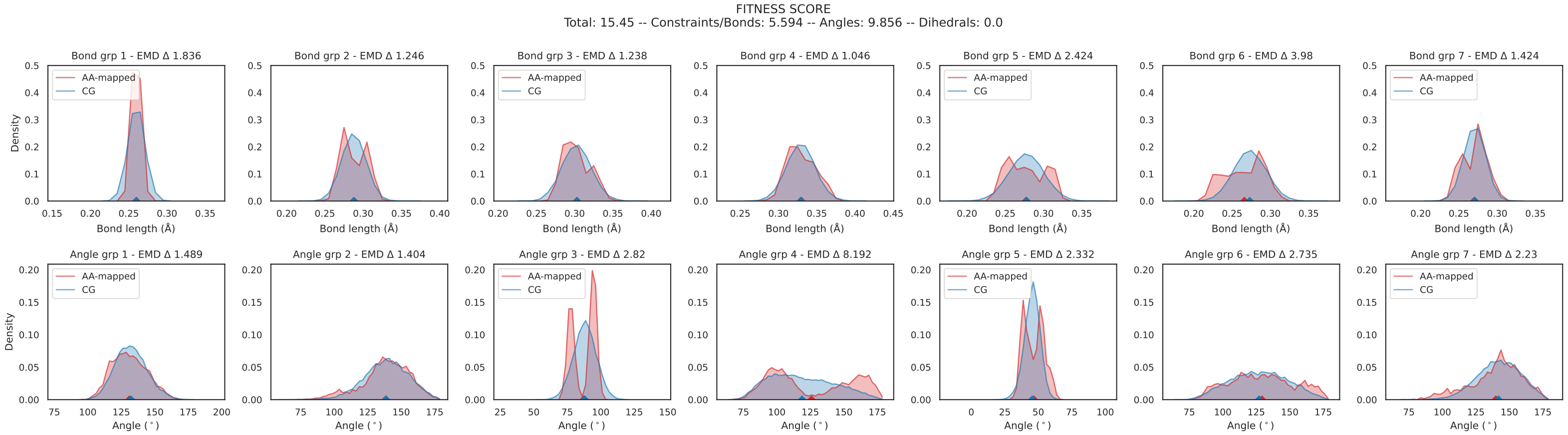

Figure S1: Bond length and angle distributions for the Finest model compared to the same distributions for the all-atomistic model.

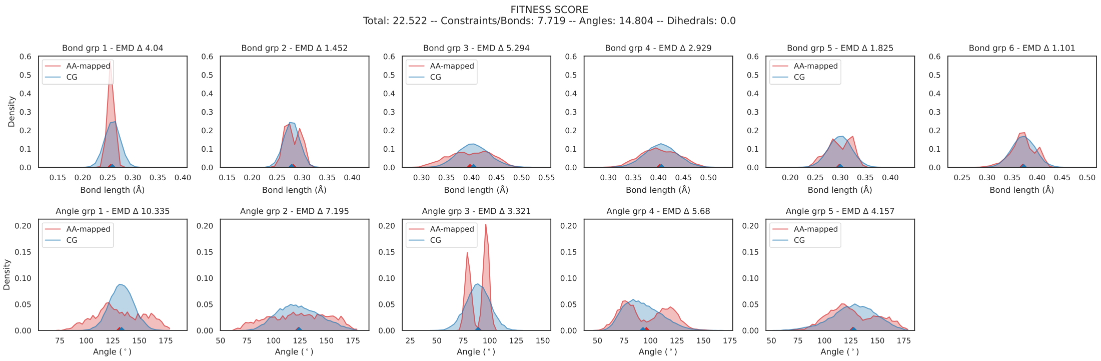

Figure S2: Bond length and angle distributions for the Mixed model compared to the same distributions for the all-atomistic model.

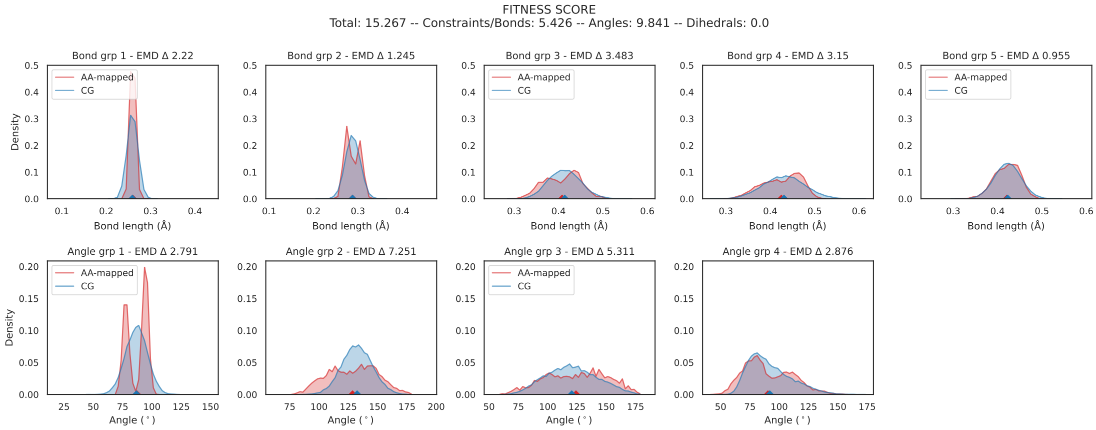

Figure S3: Bond length and angle distributions for the Coarsest model compared to the same distributions for the all-atomistic model.

S2 Dilute isotropic configurations

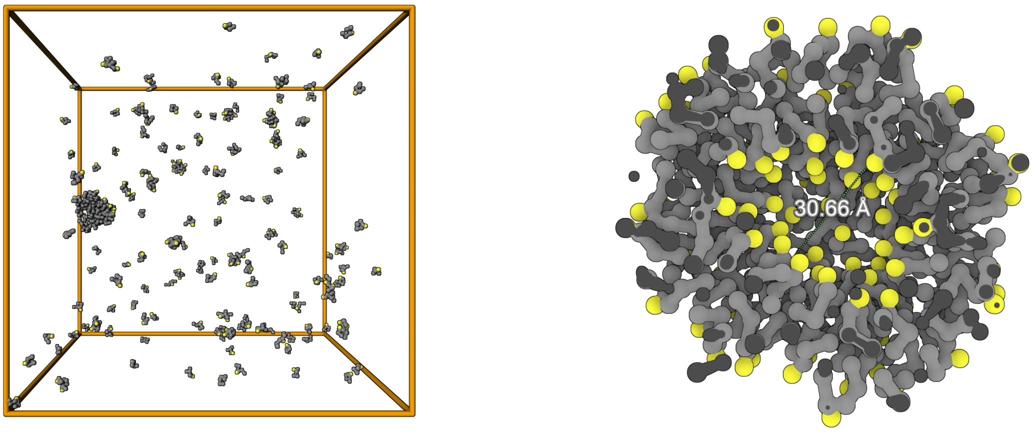

(a) 0.27 wt.% system

(b) 1 wt.% system

Figure S4: Final configurations of the Coarsest dilute isotropic systems. Yellow beads represent surfactant headgroups and grey beads represent tail groups. The box in S4a is isotropic, of side ≈ 37 nm.

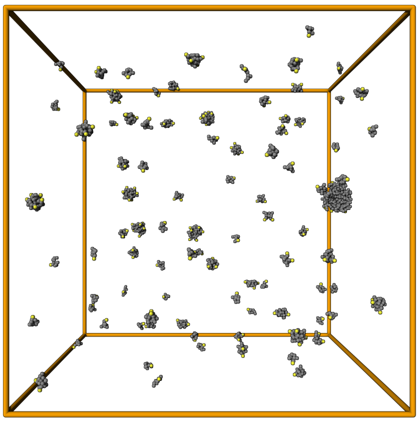

(a) 0.27 wt.% system

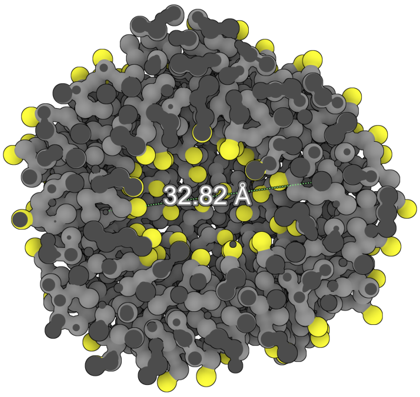

(b) 1 wt.% system

Figure S5: Final configurations of the Finest dilute isotropic systems. Yellow beads represent surfactant headgroups and grey beads represent tail groups. The box in S5a is isotropic, of side ≈ 37 nm.

S3 Bilayer box area timeseries

Figure S6 shows the total area in the *xy*-plane of the simulation boxes for each model.

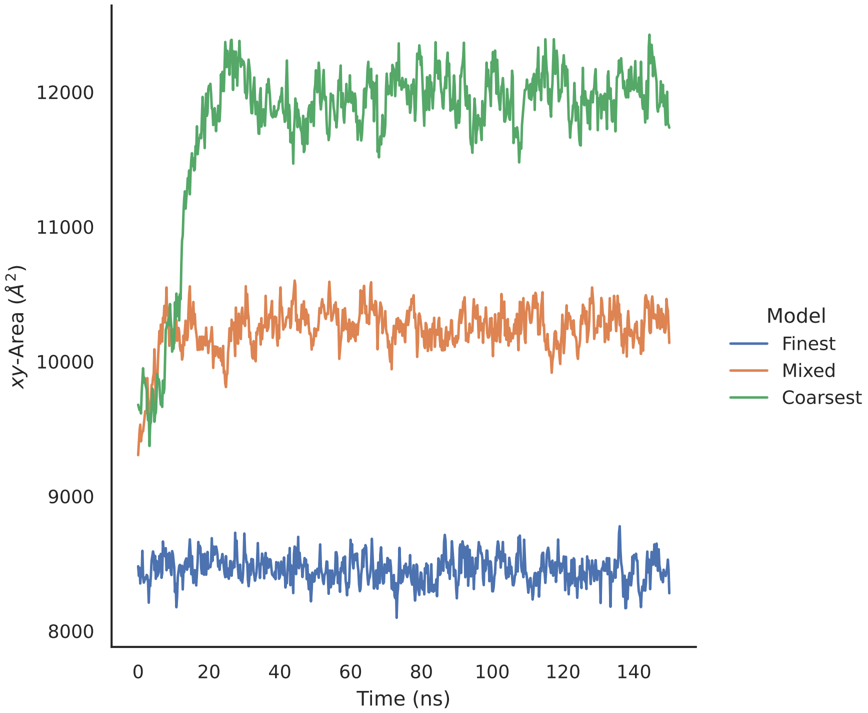

Figure S6: Total area in the *xy*-plane of the simulation box for each simulated system.

S4 Final bilayer configurations

Figure S7 shows the final configurations of the Mixed and Finest bilayer simulations, alongside the Willard-Chandler surface, computed using the Method described in the main paper.

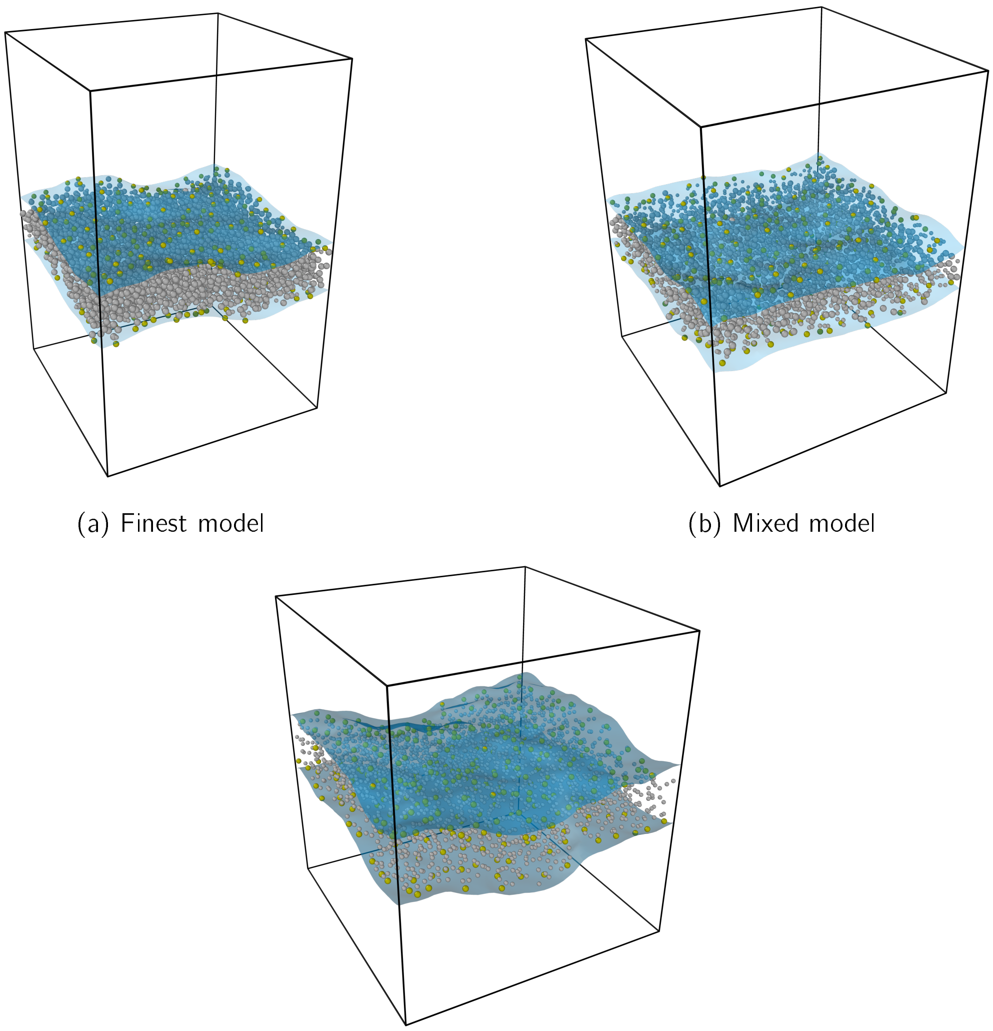

(a) Finest model

(b) Mixed model

(c) Coarsest model

Figure S7: Final configurations of the bilayer simulations. Yellow beads represent surfactant headgroups and grey beads represent tail groups. The computed Willard-Chandler surface is highlighted in blue.

References

[1] Stéphane Abel et al. "Molecular Modeling and Simulations of AOT-Water Reverse Micelles in Isooctane: Structural and Dynamic Properties". In: *The Journal of Physical Chemistry B* 108.50 (Dec. 2004), pp. 19458–19466. ISSN: 1520-6106. DOI: 10.1021/jp047138e. (Visited on 06/08/2023).

[2] Charly Empereur-Mot et al. "Swarm-CG: Automatic Parametrization of Bonded Terms in MARTINI-Based Coarse-Grained Models of Simple to Complex Molecules via Fuzzy Self-Tuning Particle Swarm Optimization". In: *ACS Omega* 5.50 (Dec. 2020), pp. 32823–32843. DOI: 10.1021/acsomega.0c05469. (Visited on 05/03/2024).
